# Relationship between macular perfusion and lesion distribution in diabetic retinopathy

**DOI:** 10.1038/s41433-024-03105-1

**Published:** 2024-05-09

**Authors:** Navid Manafi, Deniz Oncel, Aditya Verma, Giulia Corradetti, Shin Kadomoto, Alireza Mahmoudi, Ahmed Roshdy Alagorie, Naresh Kumar Yadav, Rajeev R. Pappuru, Adnan Tufail, Houri Esmaeilkhanian, Muneeswar G. Nittala, Rajiv Raman, Srinivas Sadda

**Affiliations:** 1 Doheny Eye Institute, Pasadena, CA USA; 2 Department of Ophthalmology, David Geffen School of Medicine, University of California-Los Angeles, Los Angeles, CA USA; 3 Loyola University, Chicago, Stritch School of Medicine, Chicago, IL 60153 USA; 4 Department of Ophthalmology and Visual Sciences, University of Louisville, Louisville, KY USA; 5 Department of Ophthalmology, Faculty of Medicine, Tanta University, Tanta, Egypt; 6 Smt. Kanuri Santhamma Centre for Vitreo-Retinal Diseases, L. V. Prasad Eye Institute, Kallam Anji Reddy Campus, Hyderabad, 500034 India; 7 Moorfields Eye Hospital NHS Foundation Trust, London, London, UK; 8 Shri Bhagwan Mahavir Vitreoretinal Services, Medical Research Foundation, Sankara Nethralaya, Chennai, India

**Keywords:** Retinal diseases, Vision disorders

## Abstract

**Background/objectives:**

To assess the relationship between macular vessel density metrics and foveal avascular zone (FAZ) characteristics on optical coherence tomography angiography (OCTA) and lesion distribution in eyes with diabetic retinopathy (DR).

**Subjects/methods:**

Patients with DR who underwent both Optos ultrawidefield (UWF) pseudocolor imaging and macular OCTA (Cirrus Angioplex, 6 × 6 mm) were included in this cross-sectional observational study. The distribution of DR lesions was assessed by comparing each of the peripheral ETDRS extended fields (3–7) against their corresponding ETDRS field, hence eyes were defined as either having predominantly peripheral lesions (PPL) or predominantly central lesions (PCL). En face OCTA images from the superficial and deep capillary plexuses (SCP and DCP) were then analysed using Image J software. Perfusion density (PD), vessel length density (VLD), and fractal dimensions (FD) were calculated following binarization and skeletonization of the images.

**Results:**

Out of 344 eyes, 116 (33.72%) eyes had PPL and 228 (66.28%) eyes had PCL. For all DRSS levels, VLD, PD, and FD were not significantly different between eyes with PPL and PCL. The FAZ in eyes with PPL, however, was found to be more circular in shape compared to eyes with PCL (*p* = **0.037**).

**Conclusion:**

Although the presence of PPL has been associated with a higher risk for diabetic retinopathy progression, the macular perfusion is similar in eyes with PPL and PCL. The FAZ is more circular in eyes with PPL, but the clinical relevance of this difference remains to be defined.

## Introduction

Diabetic retinopathy (DR) is an important cause of blindness in working-aged individuals worldwide [1]. With the prevalence of diabetes rising at epidemic proportions, the number of individuals with DR is expected to grow exponentially, with over 160 million patients with diabetic retinopathy expected by 2045 [1].

Although a retinal neuropathy is increasingly recognized as an important component of DR [2], most of the focus in studies of DR over the last several decades is the microangiopathy. Specifically, DR can impact the capillary circulation leading to the development of microaneurysms (ma’s), vascular leakage, and intraretinal hemorrhage (IRH) [3, 4]. Vascular leakage from ma’s and telangiectatic capillaries can result in the accumulation of diabetic macular edema (DMO) that can be associated with vision loss [3]. With progressive injury to the circulation, capillary occlusion can occur leading to areas of inner retinal microinfarction (nerve fiber layer (NFL)) infarcts known as cotton wool spots (CWS) and vascular remodeling manifesting as venous beading (VB) and intraretinal microvascular abnormalities (IRMA) [5]. With the development of progressive capillary nonperfusion and ischemia, ischemic tissues may produce vascular endothelial growth factor (VEGF) and other cytokines which can promote the development of neovascularization (NV) which if left untreated can progress to severe blindness from vitreous hemorrhage (VH) or traction retinal detachment (TRD) [6].

To describe this sequence of progression from no DR to advanced proliferative DR (PDR), several staging systems have been developed, the most common of which are the Early Treatment of Diabetic Retinopathy Study (ETDRS) DR severity scale (DRSS) and the International Classification of Diabetic Retinopathy (ICDR) [7, 8]. The ETDRS DRSS itself is based on a modification of the Airlie House Classification, which assesses the extent of specific DR lesions (ma’s, IRH, IRMA, VB, etc) in various fields of view compared to standardized reference photographs [9].

Both the ETDRS and ICDR systems, however, are based on evaluation of lesions within the seven standard ETDRS photographic fields (7SF), which only covers ~ 30% of the retinal surface area [7, 8]. This limited region of assessment was due to the limitations of the fundus imaging systems at the time of development of the ETDRS scale several decades ago [7]. Since that time, however, ultrawidefield (UWF) camera systems have become available which allow the peripheral retina to be visualized (and in total ~ 82% of the retinal surface area may be reliably accessed) [10]. Silva and colleagues have shown that more than 60% of DR lesions may be present outside the ETDRS 7SF, and in 10% of cases, considering the peripheral lesions could potentially alter the determination of the stage of DR [11]. They also observed that eyes with predominantly peripheral lesions (PPL) appeared to be associated with a higher risk of progression to PDR.

Recently, DRCR.net Protocol AA evaluated the clinical significance of PPL in a prospective fashion, and found that PPL on UWF fluorescein angiography were associated with a higher risk of DR progression, regardless of the level of DR within the ETDRS 7SF [12]. PPL on UWF pseudocolor images did not show a higher risk overall, but there was a higher risk amongst patients with higher levels of DRSS. Overall, the DRCR.net investigators concluded that this finding suggested that future DR staging systems may need to consider the presence or absence of predominantly peripheral disease.

The presence of PPL has been shown to be associated with greater peripheral nonperfusion [13], and this may explain the increased risk of progression in these cases. The status of macular perfusion in eyes with PPL, however, has not been reported. As eyes with PPL appear to have a worse prognosis, better characterization of the DR in these eyes would appear to be of importance. Thus, in this study we compare macular vessel density metrics on OCT angiography (OCTA) in DR eyes with PPL and with predominantly central lesions (PCL).

## Materials/subjects and methods

This study is a post-hoc analysis of data collected as part of a prospective observational study to evaluate the distribution of lesions in eyes with DR from India. The results of the primary analysis from the main study have been reported in a previous publication [14]. This analysis includes diabetic subjects from two of the tertiary care eye centers (Sankara Nethralaya, LV Prasad Eye Institute) where both UWF imaging and OCTA data were obtained. This study adhered to the tenets of the declaration of Helsinki and was approved by the institutional review boards (IRB) of the enrollment centers. Informed consent was obtained from all subjects.

A total of 750 eyes from 397 patients were enrolled from the two centers including 655 eyes from 345 patients from Sankara Nethralaya and 95 eyes from 52 patients at LV Prasad.

To be included in this analysis, patients had to be above 18 years of age, with type 1 or 2 diabetes mellitus, and had UWF (Optos pseudocolor) and OCTA (Cirrus Angioplex, 6 × 6 mm) imaging at the same visit. Cases with low quality images were excluded. For OCTA, exclusion criteria were a signal strength less than 7 (out of 10) or significant artifacts (motion, shadowing, vessel doubling, motion, unremoved projection). For UWF, images with poor contrast or focus or insufficient field of view to allow assessment of the DR lesion distribution by the graders were excluded.

### Image acquisition

After pupillary dilatation, UWF images were taken using the Optos Daytona Plus (Optos plc, Dunfermline, Scotland, UK). The imaging procedure included two 200° central images and four additional steered peripheral images (superior, inferior, temporal, and nasal quadrants) captured by trained photographers. Raw images were exported and submitted to the Doheny Image Reading and Research Lab (DIRRL) for analysis. The central image with the maximum gradable field was selected for the PPL analysis in this study. A montage image was not constructed, but the graders were able to view the steered images to confirm their PPL vs. PCL determination from the central image.

### Processing of UWF images

In the initial step, uncompressed high-resolution images were obtained using the V^2^ Vantage Pro software (version 2.8.0.4) [15]. Brightness and contrast were adjusted to optimize the visualization of the DR features. Stereographic projection was then applied to these images [16, 17], followed by placement of a mask representing the standard seven field ETDRS grid [9] over each image using the prototype software made available by the manufacturer (Optos plc, Dunfermline, Scotland, UK) [9].

**Fig. 1:**
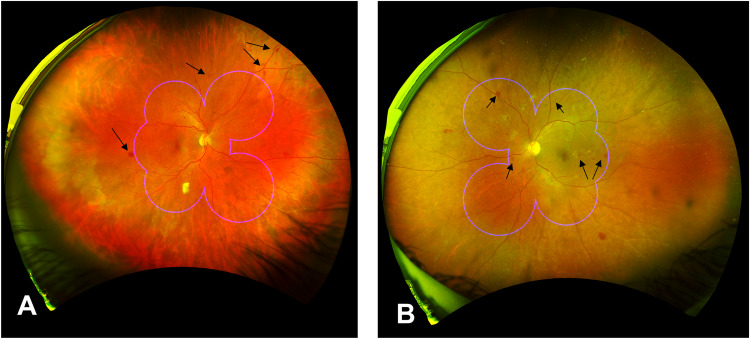
Ultra-wide field (UWF) imaging of cases with PPL and PCL. A comparison of an exemplary image with PPL (**A**; right eye) and PCL (**B**; left eye) with ETDRS field superimposed presented, respectively. Black arrows denote the lesions (retinal hemorrhages) in the specified fields. PPL predominantly peripheral lesion, PCL predominantly central lesions.

The resultant images were graded for the severity of DR, as well as the distribution of lesions. The details of the grading protocol have been published previously [14]. Briefly, the DR severity score was graded according to the ICDR grading scale within the region of the ETDRS 7SF. The distribution of DR lesions was assessed by comparing each of the peripheral ETDRS extended fields [3–7] against their corresponding ETDRS field (Fig. 1). An eye was determined to have predominantly peripheral lesions (PPL) if any peripheral field had a greater severity or extent of lesions compared to its corresponding central ETDRS field; otherwise, it was classified as having predominantly central lesions (PCL).

## OCTA grading procedure

Raw OCTA data were also exported from the Cirrus Angioplex and submitted to DIRRL for masked analysis by certified reading center OCTA graders. The automated instrument segmentation was used to generate the en face OCTA slabs for the superficial and deep capillary plexuses (SCP and DCP). Graders inspected the automated boundaries and manually corrected any segmentation errors. The corrected SCP and DCP slabs were then exported for vessel analysis using Image J (National Institutes of Health, Bethesda, Maryland, USA). Vessel density (VD) metrics analysed in this study included perfusion density (PD) and vessel length density (VLD). Vessel Length Density (VLD) is defined as the total length of perfused vasculature per unit area (mm^−1^) and Perfusion Density (PD), is defined as the total area covered by perfused vasculature per unit area (%) [18].

**Fig. 2:**
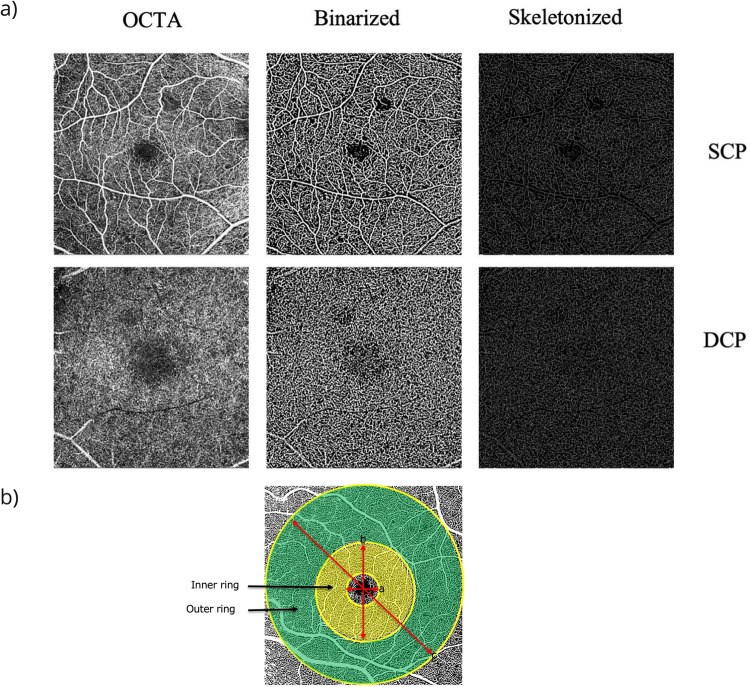
Step-by-step method of OCTA analysis. **a** OCTA images (left panels) from the superficial capillary plexus (SCP) and deep capillary plexus (DCP) following binarization (middle panels) and skeletonization (right panels). **b** OCTA 6 × 6 image areas with the EDTRS grid superimposed. The inner ring (shaded yellow) has inner and outer diameters of 1 mm and 3 mm, respectively. The outer ring (shaded green) has diameters of 3 and 6 mm, respectively.

**Fig. 3:**
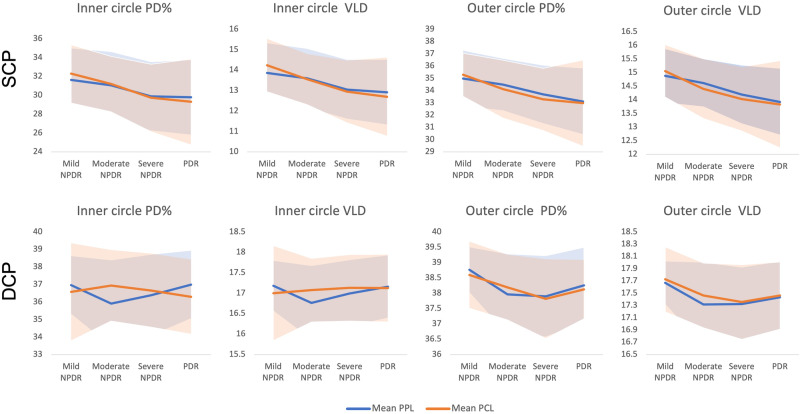
Comparison of vessel density metrics in eyes with PPL and PCL. PPL predominantly peripheral lesion, PCL predominantly central Lesions, SCP superficial capillary plexus, DCP deep capillary plexus, PD% perfusion density percentage, VLD vessel length density.

Perfusion density (PD) and vessel length density (VLD) were calculated following binarization and skeletonization, respectively. (Fig. 2) Values were computed for both the inner and outer rings of the ETDRS grid (Fig. 2) and for both the SCP and the DCP. Thus, a total of eight metrics were computed for each eye. PD and VLD of the SCP and DCP from the inner and outer rings were compared between eyes with PCL and PPL lesion distribution for varying DR severity levels (Fig. 3, Table 1).

**Table 1:** Comparison between predominantly peripheral lesion (PPL) cases and predominantly central lesions (PCL) in terms of their vascular density parameters (PD and VLD), DRSS adjusted analysis.

DRSS score	Variable	PPL	PCL	*P*-value
*Mild NPDR*	*SCP inner PD%*	31.61 ± 3.36 (19)	32.28 ± 3.06 (32)	0.47
	*SCP outer PD%*	34.98 ± 2.27 (19)	35.29 ± 1.75 (32)	0.58
	*SCP inner VLD*	13.85 ± 1.45 (19)	14.23 ± 1.28 (32)	0.34
	*SCP outer VLD*	14.87 ± 0.98 (19)	15.05 ± 0.95 (32)	0.52
	*DCP inner PD%*	36.95 ± 1.66 (19)	36.57 ± 2.77 (32)	0.58
	*DCP outer PD%*	38.76 ± 0.74 (19)	38.59 ± 1.08 (32)	0.5
	*DCP inner VLD*	17.17 ± 0.61 (19)	17.00 ± 1.16 (32)	0.53
	*DCP outer VLD*	17.66 ± 0.35 (19)	17.72 ± 0.52 (32)	0.65
*Moderate NPDR*	*SCP inner PD%*	31.04 ± 3.53 (42)	31.20 ± 2.92 (84)	0.79
	*SCP outer PD%*	34.48 ± 2.11 (42)	34.12 ± 2.36 (84)	0.4
	*SCP inner VLD*	13.59 ± 1.44 (42)	13.55 ± 1.23 (84)	0.86
	*SCP outer VLD*	14.61 ± 0.87 (42)	14.40 ± 1.08 (84)	0.26
	*DCP inner PD%*	35.91 ± 2.45 (42)	36.94 ± 2.03 (84)	**0.01**
	*DCP outer PD%*	37.96 ± 1.32 (42)	38.19 ± 1.06 (84)	0.6
	*DCP inner VLD*	16.76 ± 0.90 (42)	17.07 ± 0.77 (84)	0.52
	*DCP outer VLD*	17.31 ± 0.68 (42)	17.46 ± 0.52 (84)	0.33
*Severe NPDR*	*SCP inner PD%*	29.88 ± 3.61 (34)	29.73 ± 3.54 (53)	0.84
	*SCP outer PD%*	33.68 ± 2.36 (34)	33.28 ± 2.53 (53)	0.46
	*SCP inner VLD*	13.05 ± 1.44 (34)	12.94 ± 1.52 (53)	0.75
	*SCP outer VLD*	14.19 ± 1.07 (34)	14.02 ± 1.16 (53)	0.5
	*DCP inner PD%*	36.38 ± 2.31 (34)	36.65 ± 2.09 (53)	0.56
	*DCP outer PD%*	37.90 ± 1.32 (34)	37.81 ± 1.28 (53)	0.77
	*DCP inner VLD*	16.99 ± 0.81 (34)	17.12 ± 0.81 (53)	0.44
	*DCP outer VLD*	17.32 ± 0.59 (34)	17.35 ± 0.60 (53)	0.79
*PDR*	*SCP inner PD%*	29.78 ± 3.95 (21)	29.30 ± 4.52 (47)	0.67
	*SCP outer PD%*	33.11 ± 2.68 (21)	32.98 ± 3.51 (47)	0.88
	*SCP inner VLD*	12.90 ± 1.58 (21)	12.69 ± 1.91 (47)	0.65
	*SCP outer VLD*	13.92 ± 1.21 (21)	13.83 ± 1.59 (47)	0.81
	*DCP inner PD%*	36.98 ± 1.93 (21)	36.29 ± 2.13 (47)	0.21
	*DCP outer PD%*	38.26 ± 1.23 (21)	38.12 ± 0.95 (47)	0.62
	*DCP inner VLD*	17.15 ± 0.76 (21)	17.12 ± 0.82 (47)	0.86
	*DCP outer VLD*	17.43 ± 0.58 (21)	17.46 ± 0.54 (47)	0.84

Values are presented as mean ± standard deviation.

*SCP* superficial capillary plexus, *DCP* deep capillary plexus, *PD%* perfusion density percentage, *VLD* vessel length density, *NPDR* non-proliferative diabetic retinopathy, *PDR* proliferative diabetic retinopathy. Numbers in the brackets are number of cases. *N/A* not applicable

For assessment of FAZ characteristics, two independent masked graders (NM and AM) manually segmented the boundaries of the FAZ from the en face OCTA images of the SCP. Following segmentations, the area, maximum and minimum diameters (Lmajor and Lminor, respectively), and perimeter, axial ratio, roundness, and circularity were computed using Image J. Axial ratio is defined by the formula [(length of major axis)/(length of minor axis)] and provides an assessment of how oval or round an object is. Roundness, which is defined by the formula [4 × area/ π × (length of major axis)^2^], is a measure of how close the object conforms to a perfect circle. Circularity, which is defined by the formula [4π × area/perimeter^2^], is another measure of shape which also takes into account the smoothness of the border [19].

### Statistical analysis

The OCTA metrics from eyes with PPL and PCL were compared using an independent *t*-test. For assessing the level of significance, two-sided p-values were chosen. For statistical analysis, IBM’s SPSS software was used (IBM Corp. Released 2020. IBM SPSS Statistics for Mac, version 28.0.1.1. Armonk, NY: IBM Corp). A *P* value of <0.05 was considered statistically significant. The assessment of inter-rater agreement for analysis of FAZ characteristics was performed by measuring the intraclass correlation coefficient (ICC) model 3,1 estimates and their 95% confidence intervals based on a mean-rating (*k* = 2), absolute-agreement, 2-way mixed-effects model.

## Results

In total, 322 eyes of 248 patients from Sankara Nethralaya and 22 eyes of 18 patients from LVPEI met the quality criteria and were included in the final analysis. Out of these 344 eyes, 116 (33.7%) eyes had PPL and 228 (66.3%) eyes had PCL which is similar to the distribution in the overall cohort reported previously [14]. 175 (50.9%) eyes were left eyes, and 169 (49.1%) were right eyes. Among eyes with PPL, 19 (16.4%) had mild NPDR, 42 (36.2%) had moderate NPDR, 34 (29.3%) eyes had severe NPDR, and 21 (18.1%) had PDR. In the group with PCL, 32 (14.8%) had mild NPDR, 84 (38.9%) had moderate NPDR, 53 (24.5%) eyes had severe NPDR, and 47 (21.8%) eyes had PDR (Table 1).

Among the 12 eyes with no DR withing the 7SF, by definition all eyes with any DR lesions in the extended fields were considered as PPL (100%). Among eyes with mild NPDR (51 eyes), 19 (37.3%) had PPL and 32 (62.8%) had PCL. In the group with moderate NPDR (126 eyes), 42 (33.3%) eyes had PPL and 84 (66.7%) eyes had PCL. In eyes with severe NPDR (87 eyes), 34 (39.1%) eyes had PPL, and 53 (60.9%) eyes had PCL. Among eyes with PDR (68 eyes), 21 (30.9%) eyes had PPL and 47 (69.1%) eyes had PCL (Table 1).

### SCP layer

Comparison of eyes with PCL and PPL without considering the DR severity level showed that in the SCP layer, the inner zone of PCL cases had numerically higher PD% and VLD values, while in the outer zone, PPL cases had higher values for these parameters (Table 2). None of the differences, however, were significantly different between the two groups.

**Table 2:** Comparison of cases with predominantly peripheral lesion (PPL) and predominantly central lesions (PCL) in terms of their vascular density parameters (perfusion density percentage (PD%) and vessel length density (VLD)) in the superficial capillary plexus (SCP) and deep capillary plexus (DCP), as well as the foveal avascular zone (FAZ) characteristics.

	Variable	PPL (*N* = 116)	PCL (*N* = 228)	*P* value
vascular density parameters	*SCP inner PD%*	30.57 ± 3.63	30.72 ± 3.60	0.71
	*SCP outer PD%*	34.08 ± 2.38	33.97 ± 2.73	0.7
	*SCP inner VLD*	13.35 ± 1.49	13.40 ± 1.56	0.79
	*SCP outer VLD*	14.40 ± 1.05	14.35 ± 1.29	0.69
	*DCP inner PD%*	36.41 ± 2.22	36.73 ± 2.15	0.21
	*DCP outer PD%*	38.13 ± 1.25	38.19 ± 1.12	0.65
	*DCP inner VLD*	16.97 ± 0.81	17.11 ± 0.84	0.13
	*DCP outer VLD*	17.39 ± 0.60	17.50 ± 0.56	0.09
FAZ characteristics	Area (mm^2^)	0.490 ± 0.166	0.511 ± 0.466	0.363
	Maximum diameter (Lmajor)(mm)	0.951 ± 0.196	0.968 ± 0.358	0.360
	Minimum diameter (Lminor)(mm)	0.714 ± 0.119	0.709 ± 0.180	0.418
	Perimeter* (mm)	2.756 ± 0.564	2.797 ± 0.975	0.378
	Circularity (unitless)	0.812 ± 0.130	0.781 ± 0.130	0.061
	Roundness (unitless)	0.690 ± 0.104	0.662 ± 0.101	**0.037**
	Axial ratio (unitless)	1.335 ± 0.191	1.358 ± 0.178	0.208

Values are presented as mean ± standard deviation.

In the SCP layer, assessment of the PD% in the inner ring showed a numerically higher values in the PCL group among eyes with mild or moderate NPDR and higher values in the PPL group among eyes with severe NPDR and PDR (Table 1). However, none of these differences were statistically significant. For the inner ring, with the exception of eyes with mild NPDR, for all other DR severity levels with PPL had numerically higher PD% values in the outer ring. The differences, however, were not statistically significant.

Assessment of VLD in the inner and outer ring showed numerically higher VLD values in eyes with mild NPDR and PCL, whereas for all other DR severity levels, there was a numerically higher VLD in eyes with PPL. Again, these differences were not statistically significant. There was no difference in fractal dimensions (FD) between the group with PPL (1.54 ± 0.03) and PCL (1.54 ± 0.04) (*P*-value = 0.149).

### DCP layer

In the DCP layer, cases with PCL had numerically higher values for VLD and PD% in both the inner and outer parafoveal rings. None of the differences, however, were statistically significant (Table 1).

In the DCP layer, assessment of PD% in the inner ring showed that the cases with PPLs in the groups with mild NPDR and PDR have numerically higher PD% values, while PCL with moderate and severe NPDR have numerically higher PD% values than cases with PPL. The difference was statistically significantly different in the group with moderate NPDR, while the rest of the differences were not statistically significantly different. In the outer ring, except for the PCL cases in the moderate NPDR group, PD% was numerically higher in the PPL cases across all DR severity levels. The differences were not significantly different.

Assessment of VLD in the inner ring showed that in the mild NPDR and PDR groups, cases with PPL had numerically higher VLD values than eyes with PCL, whereas for moderate and severe NPDR eyes, cases with PPL had numerically higher VLD values. Again, none of the differences were statistically significantly different. In the outer ring, cases with PCL had numerically higher VLD values for all DR severity levels, but the differences were not significantly different. There was no difference in FD between groups with a mean FD of 1.62 ± 0.01 in both groups (*p* = 0.362).

### Foveal avascular zone (FAZ) characteristics

FAZ characteristics of both groups are summarized in Table 2. The FAZ area and perimeter were not statistically significantly different between the two groups. However, the PCL group had a larger maximum diameter (Lmajor) and smaller minimum diameter (Lminor) suggesting a more oval shape compared to the PPL group. This is corroborated by the higher roundness (*p* = 0.037) and circularity (*p* = 0.061), and lower axial ratio (*p* = 0.208) in the PPL cases. The inter-grader reliability for segmentation of the FAZ border revealed a good inter-grader reliability of 86% (ICC 3,2 = 0.855).

## Discussion

In this study, we evaluated and compared macular vessel density metrics in DR eyes with PPL or PCL lesion distribution. We observed that there were no significant differences in macular perfusion between these two groups, despite recent data showing a higher risk for DR progression in eyes with PPL.

The reason why PPL in UWF FA appears to be associated with a higher risk of DR progression, is likely related to the greater peripheral nonperfusion in these eyes. Silva et al. [13] in a cross-sectional observational study assessed the association of NPA (non-perfusion area) extent and NPI (Non-perfusion index) severity with presence of PPLs by using UWF images. They observed that on UWF images, the presence of PPLs was associated with an increase in NPA and NPI. This difference was significant even after adjusting for DR severity and diabetes duration.

In another cross-sectional observational study, Ashraf et al. [20] assessed the association of DR lesion distribution with DR severity by comparing the OCTA metrics between eyes with PPL and PCL. They found that a decrease in VD corresponded to an increase in DR severity in eyes with PCL, but such a correlation was not observed in cases with PPL. Overall, there findings suggested that VD OCTA metrics in PPL cases do not correspond to severity.

The distribution of DR severity levels was not significantly different between the PPL and PCL groups in our study. In the study by Ashraf et al. [20], however, eyes with severe NPDR/PDR were more prevalent in the cohort without PPL. When considering the VLD from the SCP layer in our study, we did observe that eyes with mild NPDR had numerically higher VLD values in the PCL group, whereas in the severe NPDR and PDR eyes, VLD was higher in the PPL group. Although these differences were not statistically significant in our study, the trend is similar to the findings of Ashraf et al. [20].

One of contrasts in the findings between the present study and the study by Ashraf et al. [20] was the numerical differences across different DR severity levels among the PPL and PCL groups. Specifically, we observed a trend for a lower VLD and PD% in the more severe DR eyes in the SCP layer for both the PCL and PPL cohorts. For both cohorts, the VLD and PD% decreased with higher DR severity levels. Although Ashraf et al. [20] observed a similar relationship in their group without PPL, this was not observed in their PPL group.

Another point of difference between the current study and the one by Ashraf et al. [20] is that the numerical reductions in the VLD and PD% values were observed more prominently in the SCP layer than the DCP layer (Fig. 3), while Ashraf et al. [20] reported no difference. It should be acknowledged, however, that the apparent differences in our study were also not significant.

The comparison of FAZ characteristics between two groups revealed a more circular shape in eyes with PPL compared to eyes with PCL, which tend to have a more elliptical shape. There was also a trend for a greater FAZ size in eyes with PCL. Taken together, the larger FAZ size and more irregular shape in eyes with PCL compared to PPL would suggest that the FAZ is more impacted in eyes with PCL. Ashraf et al. did not assess alterations in the FAZ in their study but given that they observed more severe macular perfusion alterations in eyes with PCL, one might anticipate that they would have found FAZ enlargement as well. An increased FAZ size has been associated with greater DR severity and DR progression [21], but it should be acknowledged that there can be considerable variation in FAZ size even among normal individuals [19, 22, 23], and thus the clinical relevance of differences in FAZ between eyes with PCL and PPL remains uncertain.

Our study has several limitations which should be considered when interpreting our results. First, although the imaging data were acquired in a prospective fashion, the present study is based on a posthoc analysis, and thus is still subject to ascertainment bias. Second, although the imaging data was available for all included cases, detailed demographic information including data on systemic metabolic control, disease duration, and comorbidities was not available and thus may have introduced other confounders between the groups. Third, the sample size for individual DR severity levels was relatively small, and thus comparisons between eyes with PCL and PPL may have been underpowered within specific DR severity levels. Fourth, PCL vs. PPL was determined based on the UWF pseudocolor images. The DRCR.net Protocol AA has reported that there may be discrepancies in PPL determination between color imaging and fluorescein angiography. Finally, while we have OCTA data of the macula, we did not have or consider perfusion status outside of this central region. Our study also has several strengths including the use of standardized acquisition protocols for the UWF and OCTA images and the use of experienced, certified reading center graders.

In summary, in this study we observed that macular perfusion as assessed by quantitative OCT angiography was similar in DR eyes with PCL and PPL lesion distribution. As eyes with PPL are known to have more peripheral nonperfusion, these results further highlight the disconnect between central and peripheral nonperfusion in eyes with DR.

## Summary

### What was known before

- The presence of PPL has been shown to be associated with greater peripheral nonperfusion.
- Eyes with Diabetic retinopathy and PPL lesion distribution appear to have a worse prognosis.

### What this study adds

- Macular perfusion as assessed by quantitative OCT angiography was similar in DR eyes with PCL and PPL lesion distribution.
- The FAZ in eyes with PPL is more circular in shape compared to eyes with PCL.
- The results of this study further highlight the disconnect between central and peripheral nonperfusion in eyes with DR.

## Data Availability

The data used for grading and analysis presented in this study have been gathered from centers mentioned in the paper and are securely stored and available for further access. In compliance with the Health Insurance Portability and Accountability Act (HIPAA) and to preserve patient data privacy the data used in this study is not publicly available.

## References

[1] Teo ZL, Tham YC, Yu M, Chee ML, Rim TH, Cheung N, et al. Global prevalence of diabetic retinopathy and projection of burden through 2045. Ophthalmology. 2021;128:1580–91. 33940045 10.1016/j.ophtha.2021.04.027

[2] Muc R, Saracen A, Grabska-Liberek I. Associations of diabetic retinopathy with retinal neurodegeneration on the background of diabetes mellitus. Overview of recent medical studies with an assessment of the impact on healthcare systems. Open Med. 2018;13:130–6. 10.1515/med-2018-0008 PMC5906647 29675479

[3] Murugesan N, Üstunkaya T, Feener E. Thrombosis and hemorrhage in diabetic retinopathy: a perspective from an inflammatory standpoint. Semin Thromb Hemost. 2015;41:659–64. 26305236 10.1055/s-0035-1556731 PMC4765320

[4] Vaz-Pereira S, Morais-Sarmento T, De Salvo G. Sensitivity and specificity of MultiColor imaging in detecting proliferative diabetic retinopathy. Int Ophthalmol. 2022;42:455–67. 34698967 10.1007/s10792-021-02062-y PMC8545774

[5] Sears CM, Nittala MG, Jayadev C, Verhoek M, Fleming A, Van Hemert J, et al. Comparison of subjective assessment and precise quantitative assessment of lesion distribution in diabetic retinopathy. JAMA Ophthalmol. 2018;136:365–71. 29470566 10.1001/jamaophthalmol.2018.0070 PMC5876824

[6] Demir G, Arici M, Alkin Z. Preoperative evaluation of tractional retinal detachment with B–mode ultrasonography in diabetic vitreous hemorrhage. Beyoglu Eye J. 2021;6:49–53. 35005492 10.14744/bej.2021.58561 PMC8651038

[7] Yang Z, Tan TE, Shao Y, Wong TY, Li X. Classification of diabetic retinopathy: past, present and future. Front Endocrinol (Lausanne). 2022;13:1079217. 36589807 10.3389/fendo.2022.1079217 PMC9800497

[8] Wilkinson C, Ferris FL, Klein RE, Lee PP, Agardh CD, Davis M, et al. Proposed international clinical diabetic retinopathy and diabetic macular edema disease severity scales. Ophthalmology. 2003;110:1677–82. 13129861 10.1016/S0161-6420(03)00475-5

[9] Grading diabetic retinopathy from stereoscopic color fundus photographs--an extension of the modified Airlie House classification. ETDRS report number 10. Early treatment diabetic retinopathy study research group. Ophthalmology. 1991;98:786–806. 2062513

[10] Singer M, Sagong M, van Hemert J, Kuehlewein L, Bell D, Sadda SR. Ultra-widefield imaging of the peripheral retinal vasculature in normal subjects. Ophthalmology. 2016;123:1053–9. 26896126 10.1016/j.ophtha.2016.01.022

[11] Silva PS, Cavallerano JD, Sun JK, Soliman AZ, Aiello LM, Aiello LP. Peripheral lesions identified by mydriatic ultrawide field imaging: distribution and potential impact on diabetic retinopathy severity. Ophthalmology. 2013;120:2587–95. 23778092 10.1016/j.ophtha.2013.05.004

[12] Marcus DM, Silva PS, Liu D, Aiello LP, Antoszyk A, Elman M, et al. Association of predominantly peripheral lesions on ultra-widefield imaging and the risk of diabetic retinopathy worsening over time. JAMA Ophthalmol. 2022;140:946. 35980608 10.1001/jamaophthalmol.2022.3131 PMC9389433

[13] Silva PS, Dela Cruz AJ, Ledesma MG, Van Hemert J, Radwan A, Cavallerano JD, et al. Diabetic retinopathy severity and peripheral lesions are associated with nonperfusion on ultrawide field angiography. Ophthalmology. 2015;122:2465–72. 10.1016/j.ophtha.2015.07.034. 26350546 10.1016/j.ophtha.2015.07.034

[14] Verma A, Alagorie AR, Ramasamy K, van Hemert J, Yadav N, Pappuru RR, et al. Distribution of peripheral lesions identified by mydriatic ultra-wide field fundus imaging in diabetic retinopathy. Graefe’s Arch Clin Exp Ophthalmol. 2020;258:725–33. 31989286 10.1007/s00417-020-04607-w

[15] Knickelbein JE, Hasan J, Nussenblatt RB, Sen HN. Delineation of choroidal and retinal lesions in posterior uveitis by multispectral wide-field scanning laser ophthalmoscopy. Retina. 2016;36:2213–9. 27152831 10.1097/IAE.0000000000001050

[16] Tan CS, Chew MC, van Hemert J, Singer MA, Bell D, Sadda SR. Measuring the precise area of peripheral retinal non-perfusion using ultra-widefield imaging and its correlation with the ischaemic index. Br J Ophthalmol. 2016;100:235–9. 26135013 10.1136/bjophthalmol-2015-306652

[17] Escudero-Sanz I, Navarro R. Off-axis aberrations of a wide-angle schematic eye model. J Opt Soc Am A. 1999;16:1881. 10.1364/josaa.16.001881 10435267

[18] Jung JJ, Lim SY, Chan X, Sadda SR, Hoang QV. Correlation of diabetic disease severity to degree of quadrant asymmetry in En Face OCTA Metrics. Investig Opthalmol Vis Sci. 2022;63:12. 10.1167/iovs.63.9.12 PMC9379327 35943732

[19] Shiihara H, Terasaki H, Sonoda S, Kakiuchi N, Shinohara Y, Tomita M, et al. Objective evaluation of size and shape of superficial foveal avascular zone in normal subjects by optical coherence tomography angiography. Sci Rep. 2018;8:10143. 29973663 10.1038/s41598-018-28530-7 PMC6031610

[20] Ashraf M, Sampani K, Rageh A, Silva PS, Aiello LP, Sun JK. Interaction between the distribution of diabetic retinopathy lesions and the association of optical coherence tomography angiography scans with diabetic retinopathy severity. JAMA Ophthalmol. 2020;138:1291–7. 33119083 10.1001/jamaophthalmol.2020.4516 PMC7596681

[21] Ashraf M, Sampani K, Clermont A, Abu-Qamar O, Rhee J, Silva PS, et al. Vascular density of deep, intermediate and superficial vascular plexuses are differentially affected by diabetic retinopathy severity. Investig Opthalmol Vis Sci. 2020;61:53. 10.1167/iovs.61.10.53 PMC7463180 32866267

[22] Gómez-Ulla F, Cutrin P, Santos P, Fernandez M, Abraldes M, Abalo-Lojo JM, et al. Age and gender influence on foveal avascular zone in healthy eyes. Exp Eye Res. 2019;189:107856. https://linkinghub.elsevier.com/retrieve/pii/S0014483519305895 31654619 10.1016/j.exer.2019.107856

[23] Verma A, Magesan K, Amose T, Alagorie AR, Gnanaraj R, Sadda SR, et al. Age-related assessment of foveal avascular zone and surrounding capillary networks with swept source optical coherence tomography angiography in healthy eyes. Eye. 2022;36:1857–64. https://www.nature.com/articles/s41433-022-02146-8 35948688 10.1038/s41433-022-02146-8 PMC9500041

